# Essential Role of Keap1-Nrf2 Signaling in Mood Disorders: Overview and Future Perspective

**DOI:** 10.3389/fphar.2018.01182

**Published:** 2018-10-16

**Authors:** Kenji Hashimoto

**Affiliations:** Division of Clinical Neuroscience, Chiba University Center for Forensic Mental Health, Chiba, Japan

**Keywords:** brain-derived neurotrophic factor, glucoraphanin, keap1, Nrf2, nutrition, stress resilience, sulforaphane, TrkB

## Abstract

Depression is one of the most common mood disorders with a high rate of relapse. Accumulating evidence suggests that the transcription factor Kelch-like erythroid cell-derived protein with CNC homology (ECH)-associated protein 1 (Keap1)-Nuclear factor (erythroid-derived 2)-like 2 (Nrf2) system plays a key role in inflammation which is involved in depression. Preclinical studies demonstrated that the protein expressions of Keap1 and Nrf2 in the prefrontal cortex (PFC), CA3 and dentate gyrus (DG) of hippocampus in mice with depression-like phenotype were lower than control mice. In the learned helplessness paradigm, the protein levels of Keap1 and Nrf2 in the PFC and DG of hippocampus from rats with depression-like phenotype were also lower than control and resilient rats. Furthermore, rodents with depression-like phenotype have higher levels of pro-inflammatory cytokines. Interestingly, *Nrf2* knock-out (KO) mice exhibit depression-like phenotype, and higher serum levels of pro-inflammatory cytokines compared with wild-type mice. Furthermore, *Nrf2* KO mice have lower expression of brain-derived neurotrophic factor (BDNF) in the PFC, and CA3 and DG of hippocampus compared to wild-type mice. 7,8-Dihydroxyflavone, a TrkB agonist, showed antidepressant effects in *Nrf2* KO mice, by stimulating BDNF-TrkB in the PFC, CA3, and DG. Pretreatment with sulforaphane, a naturally occurring Nrf2 activator, prevented depression-like phenotype in mice after inflammation, or chronic social defeat stress. Interestingly, dietary intake of 0.1% glucoraphanin (a precursor of sulforaphane) containing food during juvenile and adolescent stages of mice could prevent depression-like phenotype in adulthood after chronic social defeat stress. Moreover, the protein expressions of Keap1 and Nrf2 in the parietal cortex from major depressive disorder and bipolar disorder were lower than controls. These findings suggest that Keap1-Nrf2 system plays a key role in the stress resilience which is involved in the pathophysiology of mood disorders. It is, therefore, possible that dietary intake of cruciferous vegetables including glucoraphanin (or SFN) may prevent or minimize relapse from remission, induced by stress and/or inflammation in depressed patients. In the review, the author would like to discuss the role of Keap1-Nrf2 system in mood disorders.

## Introduction

Depression, one of the most common psychiatric disorders in the world, is a mood disorder with a high rate of relapse. The World Health Organization (WHO) estimates that more than 320 million individuals of all ages suffer from depression, highlighting this disease as a major contributor to the global burden of disease (World Health Organization [WHO], 2017). Although the precise mechanisms underlying the pathophysiology of depression are currently unknown, accumulating evidence implicate inflammatory processes in the pathophysiology of depression (Dantzer et al., 2008; Hashimoto, 2009; Miller et al., 2009; Raison et al., 2010; Hashimoto, 2015; Mechawar and Savitz, 2016; Miller and Raison, 2016; Zhang et al., 2016; Miller et al., 2017). Meta-analysis demonstrated higher levels of pro-inflammatory cytokines in the blood of drug-free or medicated depressed patients compared to healthy controls (Dowlati et al., 2010; Young et al., 2014; Haapakoski et al., 2015; Köhler et al., 2018). Studies demonstrated elevated gene expression of pro-inflammatory cytokines in the postmortem brain samples from patients with a history of depression (Dean et al., 2010; Shelton et al., 2011). Collectively, it is likely that inflammation plays a key role in the pathophysiology of depression.

Over the past decade, there has been increasing interest in the potential benefits of early intervention for mood disorders. Several lines of evidence suggest that nutrition has a high impact on the development of depression (Lin et al., 2010; Murakami and Sasaki, 2010; Bazinet and Layé, 2014; Mello et al., 2014; El-Behadli et al., 2015; Opie et al., 2015; Lin et al., 2017; Hsu et al., 2018; Wang et al., 2018). Recent meta-analyses demonstrated that high intake of fruit, vegetables, fish, and whole grains are associated with a lower risk of depression (Lai et al., 2014; Liu et al., 2016; Saghafian et al., 2018).

In the review, the author would like to discuss the role of Keap1 [Kelch-like erythroid cell-derived protein with CNC homology [ECH]-associated protein 1)]-Nrf2 [Nuclear factor (erythroid 2-derived)-like 2] system in the pathophysiology of depression since Keap1-Nrf2 system plays a key role in inflammation. Furthermore, we also refer to the clinical significance of natural Nrf2 activator sulforaphane (SFN) as nutritional intervention for mood disorders.

## Keap1-Nrf2 System

Nrf2 is the transcription factor with a key role in cellular defense against oxidative stress. It binds to the antioxidant response elements (ARE) located in the promoter region of genes encoding many phase II detoxifying or antioxidant enzymes and related stress-responsive proteins (Kobayashi et al., 2013; Ma, 2013; Suzuki et al., 2013a; Suzuki and Yamamoto, 2015; Yamamoto et al., 2018). Under normal conditions, Nrf2 is repressed by Keap1, which is an adaptor protein for the degradation of Nrf2 (Suzuki et al., 2013a; Suzuki and Yamamoto, 2015). During oxidative stress, Nrf2 is de-repressed and activates the transcription of protective genes (Suzuki et al., 2013a; Suzuki and Yamamoto, 2015). Importantly, the Keap1-Nrf2 system plays a role in inflammation-associated pathogenesis (Kobayashi et al., 2013; Suzuki et al., 2013a; O’Connell and Hayes, 2015; Suzuki and Yamamoto, 2015; Wardyn et al., 2015; Yamamoto et al., 2018). In cancer cells, Nrf2 activation is beneficial and deleterious for the cancer-bearing host, depending on the time (initiation, promotion, and metastasis) and place (cancer cells or microenvironment) (Yamamoto et al., 2018).

## Nrf2 Activators

**FIGURE 1:**
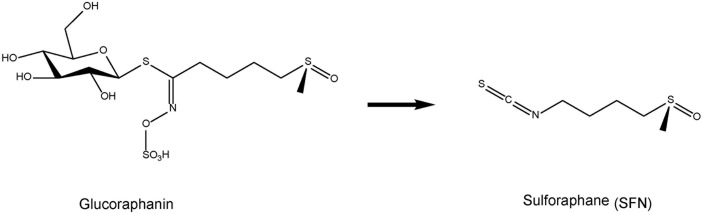
Chemical structure of sulphoraphane (SFN) and its precursor glucoraphanin. Cruciferous vegetables contain glucoraphanin, a glucosinolate derivative of sulforaphane (SFN).

Based on the role of Nrf2 in the prevention of a wide variety of pathological conditions, great efforts have been made to isolate from natural sources or develop potent and specific Nrf2 activators (Yamamoto et al., 2018). The potent anti-inflammatory and naturally occurring compound sulforaphane (SFN: 1-isothiocyanato-4-methylsulfinylbutane) is an organosulfur compound derived from a glucosinolate precursor glucoraphanin (a glucosinolate, or β-thioglucoside-*N*-hydroxysulfate) (**Figure 1**) found in cruciferous vegetables, such as broccoli sprout (Zhang et al., 1992; Fahey et al., 1997; Kwak and Kensler, 2010; Kensler et al., 2013; Fahey et al., 2015). It is well known that glucoraphanin can be converted to SFN by the endogenous enzyme, myrosinase (Fahey et al., 2015). Beneficial effect by SFN is thought to be mediated via activation of the Nrf2 pathway with subsequent up-regulation of phase II detoxification enzymes and antioxidant proteins, through ARE (Suzuki et al., 2013a; Suzuki and Yamamoto, 2015).

**FIGURE 2:**
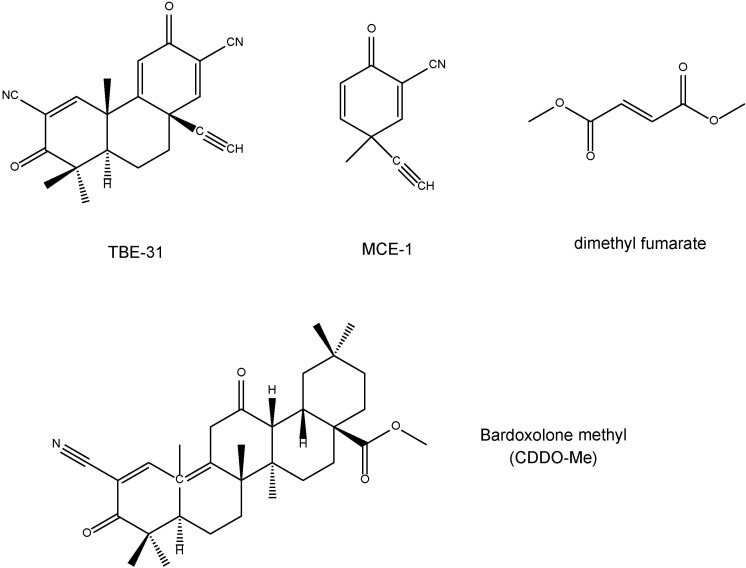
Chemical structure of Nrf2 activators (TBE-31, MCE-1, dimethyl fumarate, bardoxolone methyl).

TBE-31 [(±)-(4bS,8aR,10aS)-10a-ethynyl-4b,8,8-trimethyl-3,7-dioxo-3,4b,7,8,8a,9,10,10a-octahydrophenanthrene-2,6-dicarbonitrile] and MCE-1 [(±)-3-ethynyl-3-methyl-6-oxocyclohexa-1,4-dienecarbonitrile] are the novel Nrf2 activators (Honda et al., 2007; Dinkova-Kostova et al., 2010; Honda et al., 2011; Kostov et al., 2015; **Figure 2**). Dimethyl fumarate (**Figure 2**) is a new oral drug for the treatment of multiple sclerosis, and has neuroprotective effects via Nrf2-dependent antioxidant response (Al-Jaderi and Maghazachi, 2016; Mills et al., 2018). Bardoxolone methyl, the C-28 methyl ester of 2-cyano-3,12-dioxoolean-1,9-dien-28-oic acid (CDDO) known as CDDO-Me (**Figure 2**), is one of the derivatives of synthetic triterpenoids. Bardoxolone methyl has been used for the treatment of cancer (including leukemia and solid tumors), chronic kidney disease, and other diseases (Wang et al., 2014). Clinical trial of bardoxolone methyl is undergoing for diabetic nephropathy in Japan (Yamamoto et al., 2018), although its development was paused in the United States due to the occurrence of cardiac complications in patients with end-stage renal disease (Pergola et al., 2011; de Zeeuw et al., 2013).

## Effects of Nrf2 Activators on Neurite Outgrowth

The neuronal plasticity, including neurite outgrowth and neuroprotection, plays crucial role in the beneficial effect of therapeutic drugs in cellular level (Lu and Dwyer, 2005; Williams and Dwyer, 2009). Yao et al. (2016b) reported that SFN increased the number of cell with neurite outgrowth in PC12 cells. Furthermore, the potentiating effects of SFN on NGF-induced neurite outgrowth were blocked by treatment with *Nrf2* siRNA, but not the negative control (Yao et al., 2016b), suggesting that SFN can potentiate NGF-induced neurite outgrowth via activation of Nrf2.

Yao et al. (2016a) reported that TBE-31 and MCE-1 also potentiated NGF-induced neurite outgrowth in PC12 cells. The *Nrf2* siRNA blocked the potentiating effects of TBE-31 and MCE-1 on neurite outgrowth in PC12 cells. Astragaloside IV is also reported to attenuate lead-induced inhibition of neurite outgrowth through activation of Akt-dependent Nrf2 pathway (Yu et al., 2017). Collectively, it is likely that Nrf2 activators can promote neurite outgrowth through Nrf2 activation (Yang et al., 2015c; Yao et al., 2016a,b).

## Alterations in Keap1-Nrf2 Signaling in Rodents with Depression-Like Phenotype

Chronic social defeat stress (CSDS) model has been used widely as an animal model of depression (Krishnan and Nestler, 2008; Nestler and Hyman, 2010; Golden et al., 2011). Susceptible mice with depression-like phenotype after CSDS have higher blood levels of pro-inflammatory cytokines [e.g., tumor necrosis factor (TNF)-α, interleukin (IL)-6, IL-10, and IL-1β] (Zhang et al., 2017a).

Western blot analysis showed that protein levels of Keap1 and Nrf2 in the CA3, DG, and PFC from mice with depression-like phenotype were significantly lower than those of control mice (Yao et al., 2016b). In contrast, protein levels of Keap1 and Nrf2 in the CA1 and NAc were not different compared to control (Yao et al., 2016b). These findings suggest that lower levels of Keap1 and Nrf2 in the CA3, DG, and PFC may be involved in depression-like phenotypes after CSDS.

Learned helplessness (LH) model has been also used as an animal model of depression (Krishnan and Nestler, 2008). In the LH paradigm, approximately 20–40% rats are resilient to inescapable stress (Yang et al., 2015a,b; Yang et al., 2016). LH (susceptible) rats have higher blood levels of IL-6 than control and resilient rats (Yang et al., 2015a), suggesting that peripheral inflammation may contribute to resilience versus susceptibility to stress. Protein levels of Keap1 and Nrf2 in the PFC and DG of hippocampus from LH (susceptible) rats were lower than control and non-LH (resilient) rats (Zhang et al., 2018). These results suggest that Keap1-Nrf2 signaling may contribute to stress resilience which is involved in the pathophysiology of major psychiatric disorders (Zhang et al., 2018).

## Alterations in Keap1-Nrf2 Signaling in Samples from Patients with Major Depressive Disorder

Major depressive disorder (MDD) patients (*n* = 30) exhibited higher levels of Nrf2 and its regulator Keap1, as well as NF-κB in the cytoplasm of peripheral blood mononuclear cells compared to healthy controls (*n* = 35), suggesting that depression may be characterized by up-regulation of the transcription factor Keap1-Nrf2 (Lukic et al., 2014). Using genome-wide transcriptional profiling and promoter-based bioinformatic strategies, Mellon et al. (2016) measured leukocyte transcription factor (TF) activity in leukocytes from un-medicated MDD subjects (*n* = 20) versus age- and sex-matched healthy controls (*n* = 20). In leukocytes from un-medicated MDD subjects, the bioinformatic analysis showed an increased transcriptional activity of cAMP response element-binding/activating TF (CREB/ATF) and increased activity of TFs associated with Nrf2. Antidepressant therapy for 8 weeks was associated with significant reductions in depressive symptoms and reduced activity of Nrf2, but not in CREB/ATF activity. By contrast, other transcriptional regulation pathways, including nuclear factor kappa-B cells (NF-κB), early growth response proteins 1-4 (EGR1-4), the glucocorticoid receptor, and interferon-responsive TFs, showed either no difference as a function of disease or treatment. These results suggest that Nrf2 signaling may contribute to MDD by activating immune cell transcriptome dynamics that ultimately may influence motivational and affective processes via circulating mediators (Mellon et al., 2016).

Postmortem tissue from patients with psychiatric disorders is an underutilized substance that may be used to translate genetic and/or preclinical studies (Hashimoto et al., 2007; McCullumsmith et al., 2013; Mechawar and Savitz, 2016; Yang et al., 2017). A study using postmortem brain samples showed decreased expressions of Keap1 and Nrf2 in the parietal cortex from patients with MDD and bipolar disorder compared to control group (Zhang et al., 2018). A recent study showed the reduced (-21%) expression of Nrf2 in the dorsolateral prefrontal cortex from MDD patients (Martín-Hernández et al., 2018). These results suggest that decreased Keap1-Nrf2 signaling plays a key role in the pathophysiology of mood disorders such as MDD and bipolar disorder (Zhang et al., 2018).

## Single Nucleotide Polymorphisms in the *Nrf2* Promoter Gene in Humans

**FIGURE 3:**
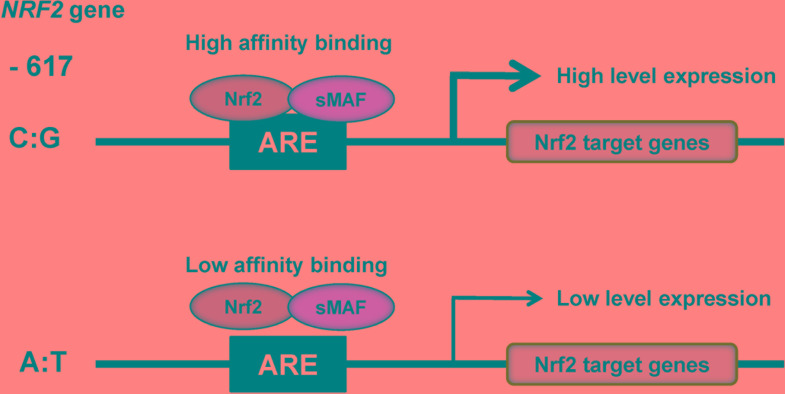
SNP (rs6721961) in the promotor region of *NRF2* gene. The SNP (rs6721961) in the promotor of *NRF2* gene alters the transcription level of *NRF2* gene, resulting in alterations in the expression of *NRF2* target genes. The NRF2/sMAF (small MAF) protein complex regulates the oxidative stress response by occupying *cis*-acting enhancers containing an antioxidant response element (ARE). A slight modification of Yamamoto et al. (2018).

The NRF2 activity in humans is regulated through protein stabilization, primarily by KEAP1, but is also regulated at the transcriptional level (Yamamoto et al., 2018). In humans, a *NRF2* promotor single nucleotide polymorphism (SNP: rs6721961) located 617 bp by upstream from the transcription start site lowers the level of *NRF2* transcription (Yamamoto et al., 2006; Yamamoto et al., 2018; **Figure 3**). Luciferase assays showed that polymorphism at position -617 (C to A) affect basal levels of NRF2, thereby resulting in attenuation of ARE-mediated gene transcription (Marzec et al., 2007; **Figure 3**). Interestingly, subjects who possess this SNP are more susceptible to acute lung injury and related diseases (Marzec et al., 2007), and this SNP is also found to correlate with the incidence of non-small cell lung cancer (Suzuki et al., 2013b). In addition, ethnic difference of this SNP is also reported (Marzec et al., 2007). Therefore, it is of interest to study whether this SNP can affect susceptibility to mood disorders.

## Depression-Like Phenotypes in *Nrf2* KO Mice

Serum levels of TNF-α, IL-6, IL-10, and IL-1β in the *Nrf2* KO mice were significantly higher than those of wild-type (WT) mice, suggesting that *Nrf2* KO mice have inflammation (Yao et al., 2016b). In the tail-suspension test (TST) and forced swimming test (FST), the immobility times of TST and FST in *Nrf2* KO mice were higher than those of WT mice. In the 1% sucrose preference test (SPT), the sucrose preference of *Nrf2* KO mice was lower than that of WT mice, suggesting that *Nrf2* KO mice have anhedonia. Furthermore, brain-derived neurotrophic factor (BDNF) and its receptor TrkB signaling in the CA3, DG and PFC of *Nrf2* KO mice were lower than those of WT mice. Moreover, protein levels of AMPA receptor 1 (GluA1) and postsynaptic density protein 95 (PSD-95) in the CA3, DG, and PFC of KO mice were lower than those of WT mice. Interestingly, 7,8-dihydroxyflavone (a TrkB agonist) produced antidepressant effects in *Nrf2* KO mice, by stimulating TrkB in the PFC, CA3, and DG (Yao et al., 2016b). Furthermore, the anti-inflammatory drug rofecoxib reversed depression-like behaviors in *Nrf2* KO mice (Martín-de-Saavedra et al., 2013). In addition, chronic treatment with the selective serotonin reuptake inhibitor (SSRI) fluoxetine increased BDNF in cortex and hippocampus of corticosterone-treated *Nrf2* KO mice (Mendez-David et al., 2015), suggesting that Nrf2 signaling contributes to fluoxetine-induced neuroprotection. These all findings suggest that Nrf2 plays a key role in the depression-like phenotypes in rodents through potent anti-inflammatory action. Collectively, it is likely that *Nrf2* KO mice show depression-like phenotypes through inflammation, decreased BDNF-TrkB signaling and synaptogenesis (**Figure 5**; Yao et al., 2016b).

In contrast, Bouvier et al. (2017) reported that *Nrf2* KO mice did not display a depression-like phenotype although the KO mice were characterized by oxidative stress and by anatomical alterations in hippocampal CA3 pyramidal cells. However, when exposed to 3 weeks of chronic mild stress, *Nrf2* KO mice developed depression-like phenotypes which were prevented by pretreatment with antioxidant (Bouvier et al., 2017). This study also suggests the role of Nrf2-dependent persistent oxidative stress in stress-induced vulnerability to depression.

## Antidepressant Effects of Nrf2 Activators in the Rodent Models of Depression

When lipopolysaccharide (LPS), the bacterial endotoxin, is administered to rodents, depression-like behaviors are observed 24 h after inflammation (Dantzer et al., 2008; O’Connor et al., 2009; Zhang et al., 2014; Zhang et al., 2016). Pretreatment with antidepressants, such as SSRIs and serotonin and norepinephrine reuptake inhibitors (SNRIs), can prevent depression-like behavior and alterations in serum pro-inflammatory cytokines, such as TNF-α, induced by LPS administration (Ohgi et al., 2013; Yao et al., 2015; Dong et al., 2016). These all findings suggest that inflammation is associated with depression, and that anti-inflammatory drugs could ameliorate depressive symptoms in patients with depression.

Pretreatment with SFN significantly blocked an increase in the serum TNF-α level after a single administration of LPS (Zhang et al., 2017b). Furthermore, SFN significantly potentiated increased serum levels of IL-10 after LPS administration. SFN attenuated an increase of the immobility time of TST and FST after LPS administration. In addition, SFN recovered to control levels for LPS-induced alterations in the proteins such as BDNF, PSD-95 and GluA1, and dendritic spine density in the brain regions (Zhang et al., 2017b). Furthermore, TBE-31 or MCE-1 attenuated an increase in serum levels of TNF-α after LPS administration. Administration of TBE-31 or MCE-1 attenuated an increase in the immobility time of TST and FST after LPS administration (Yao et al., 2016a).

Pretreatment with SFN attenuated the decreased social avoidance time and sucrose preference in CSDS model. Furthermore, SFN could attenuate the decreased levels of Nrf2 and Keap1 proteins in the PFC and hippocampus of mice with depression-like phenotype (Yao et al., 2016b). Li et al. (2018) reported that decreased Keap1-Nrf2 signaling in the PFC, hippocampus and skeletal muscle may contribute to anhedonia susceptibility after spared nerve injury (SNI), and that SFN exerts beneficial effects in SNI rats by normalization of decreased Keap1-Nrf2 signaling. These results suggest that Keap1-Nrf2 signaling plays a role in depression, and that SFN is prophylactic compound which can stimulate Keap1-Nrf2 signaling pathway (Yao et al., 2016b; Zhang et al., 2017b; Li et al., 2018). Taken all together, it is likely that the Nrf2 activators such as SFN, TBE-31, and MCE-1 might be potential prophylactic or therapeutic drugs for inflammation (or stress)-related depression (Yao et al., 2016a,b; Zhang et al., 2016, 2017b; Li et al., 2018).

## Effects of Dietary Intake of SFN Precursor in the CSDS Model of Depression

SFN is produced in the body from its precursor glucoraphanin which is involved in cruciferous vegetables. Previously, we demonstrated that dietary intake of 0.1% glucoraphanin –rich food during juvenile and adolescence prevented phencyclidine-induced cognitive deficits and loss of parvalbumin (PV)-positive cells in the PFC at adulthood (Shirai et al., 2015). Furthermore, we also reported that dietary intake of 0.1% glucoraphanin –rich food during juvenile and adolescence prevented the onset of psychosis in the adult offspring after maternal immune activation (Matsuura et al., 2018). These findings suggest that dietary-intake of glucoraphanin-rich vegetables in high-risk psychosis subjects might prevent the transition to psychosis in young adulthood (Hashimoto, 2014; Shirai et al., 2015; Matsuura et al., 2018).

Interestingly, dietary intake of 0.1% glucoraphanin containing food during juvenile and adolescent stages could prevent the depression-like phenotype in adulthood after CSDS (Yao et al., 2016b). Thus, the dietary intake of 0.1% glucoraphanin containing food during juvenile and adolescent periods could confer stress resilience in adulthood.

## Clinical Study of SFN in Patients with Healthy Subjects, and Neurodevelopmental Disorders

Sedlak et al. (2018) reported that SFN increased blood glutathione (GSH) levels in healthy human subjects following 7 days of daily oral administration. Furthermore, a significant positive correlation between blood and thalamic GSH post- and pre-SFN treatment ratios was observed, in addition to a consistent increase in brain GSH levels in response to treatment. This study suggests the value of exploring relationships between peripheral GSH and clinical/neuropsychological measures, as well as the influences SFN has on functional measures that are altered in neuropsychiatric disorders.

A randomized, double-blinded, placebo-controlled study showed that SFN-rich broccoli sprout extract could improve social interaction, abnormal behavior and verbal communication in young male subjects with autism spectrum disorder (Singh et al., 2014; Lynch et al., 2017). In addition, a pilot study showed that supplementation with glucoraphanin-rich broccoli sprout extract for 8 weeks was effective in treating cognitive impairment in medicated patients with schizophrenia (Shiina et al., 2015). Collectively, it is likely that SFN would be potential therapeutic compound for neurodevelopmental disorders.

## Role of Nrf2 in the Mechanisms of Antidepressant Action for Other Potential Compounds

Cilostazol is used in the treatment of the symptoms of intermittent claudication in patients with peripheral vascular disease. In the chronic restraint stress (CRS) model, cilostazol prevented depressive-like behaviors (Abuelezz and Hendawy, 2018). Furthermore, cilostazol modulated the Nrf2 protein and heme oxygenase-1 and NAD(P)H: quinone oxidoreductase-1 gene expression in the hippocampus of CRS rats. These findings suggest that cilostazol has the prophylactic antidepressant effect by preventing oxidative stress by stimulation of redox defense mechanisms mediated through the Nrf2 pathway (Abuelezz and Hendawy, 2018).

Dl-3-n-Butylphthalide (NBP), a small molecule compound extracted from the seeds of *Apium graveolens*, was approved by the State Food and Drug Administration of China for treating ischemic stroke (Abdoulaye and Yi, 2016). NBP attenuated the depression-like behaviors and increased expression of pro-inflammatory cytokines (e.g., IL-1β and IL-6) in rats. In addition with the anti-inflammation action, NBP reduced LPS-induced oxidative stress reactions in the hippocampus and enhanced Nrf2-targeted signals (Yang et al., 2018).

A randomized, double-blind, placebo-controlled trial showed that NBP showed greater effects than placebo on Alzheimer’s disease assessment scale-cognitive subscale (ADAS-cog) and clinician’s interview-based impression of change plus caregiver input (CIBIC-plus). NBP-related adverse events were uncommon and primarily consisted of mild gastrointestinal symptoms (Jia et al., 2016). Therefore, it is of interest to examine whether NBP can improve depressive symptoms in depressed patients.

## Nrf2 Inhibitors

**FIGURE 4:**
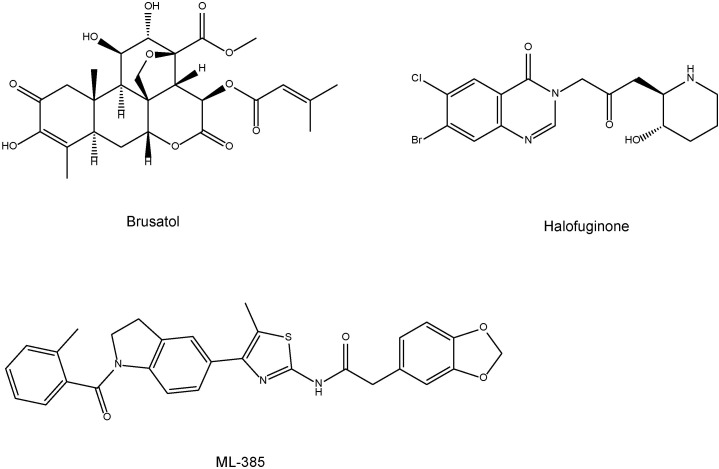
Chemical structure of Nrf2 inhibitors (brustal, halofuginone, ML-385).

Compared with Nrf2 activators, the development of Nrf2 inhibitors is in its infancy (Yamamoto et al., 2018). For example, cancers with persistent activation of Nrf2 exhibit high dependency on Nrf2 function for drug resistance and cell proliferation (Yamamoto et al., 2018). The plant-based product brusatol (**Figure 4**) decreases the protein levels of Nrf2 and sensitizes cancer cells to chemotherapy and radiotherapy (Ren et al., 2011). Another Nrf2 inhibitor halofuginone (**Figure 4**) is a synthetic halogenated derivative of febrifugine, a natural quinazolinone alkaloid which can be found in the Chinese herb *Dichroa febrifuga*. Halofuginone exerts a chemosensitizing effect on cancer cells exhibiting constitutive Nrf2 stabilization (Tsuchida et al., 2017). In addition, Singh et al. (2016) demonstrated that ML385 [*N*-[4-[2,3-dihydro-1-(2-methylbenzoyl)-1H-indol-5-yl]-5-methyl-2-thiazolyl]-1,3-benzodioxole-5-acetamide] (**Figure 4**) is a novel and specific Nrf2 inhibitor. Therefore, it is of interest to study whether these Nrf2 inhibitors can affect depression-like phenotypes in rodents.

## Conclusion Remarks and Future Perspective

**FIGURE 5:**
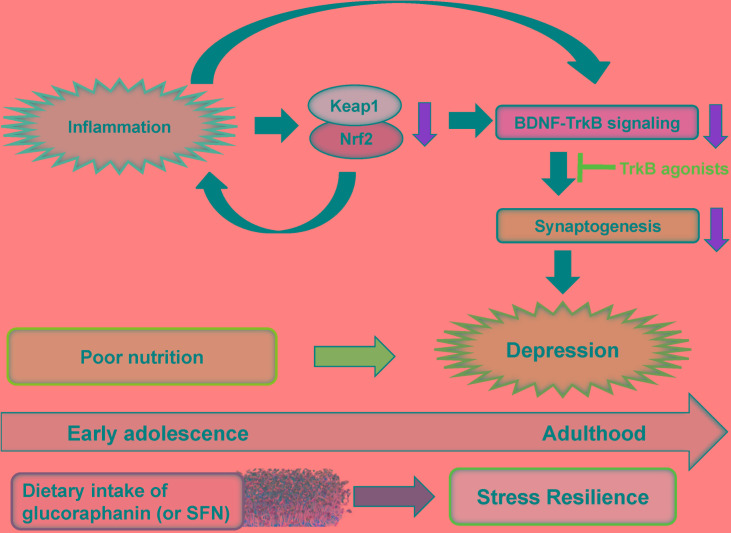
Proposed hypothesis of role of Keap1-Nrf2 system in depression. Inflammation causes decreases of Keap1 and Nrf2 expression in the prefrontal cortex and hippocampus. Subsequently, inflammation-induced decreases in Keap1 and Nrf2 proteins can cause the decreased BDNF-TrkB signaling and synaptogenesis, resulting in depression-like phenotype. TrkB agonists might have antidepressant actions. Dietary intake of glucoraphanin (or SFN) in cruciferous vegetables during early adolescence may confer to stress resilience at adulthood whereas poor nutrition may play a role in the onset of depression by stress or inflammation.

Rodents with depression-like phenotype have higher blood levels of pro-inflammatory cytokines, suggesting that inflammation plays a role in depression-like phenotype in rodents. Furthermore, rodents with depression-like phenotype have lower expression of Keap1 and Nrf2 in the PFC and hippocampus (Yao et al., 2016b; Zhang et al., 2017b). Interestingly, we found decreased expression of Keap1 and Nrf2 in the parietal cortex from patients with MDD and bipolar disorder (Zhang et al., 2018). Given the essential role of BDNF-TrkB signaling in depression (Nestler et al., 2002; Hashimoto et al., 2004; Duman and Monteggia, 2006; Hashimoto, 2010; Zhang et al., 2016), inflammation (or stress)-induced reduction of Keap1-Nrf2 system may contribute to decreased BDNF-TrkB signaling and synaptogenesis, resulting in depression-like phenotypes (**Figure 5**). It is noteworthy that TrkB agonist 7,8-DHF has antidepressant effects in *Nrf2* KO mice (Yao et al., 2016b), LPS-treated mice (Zhang et al., 2014) and CSDS susceptible mice (Zhang et al., 2015), suggesting a possible link between Keap1-Nrf2 system and BDNF-TrkB signaling (Mendez-David et al., 2015; Yao et al., 2016b; Zhang et al., 2017b; Li et al., 2018; **Figure 5**).

Nutritional status during early adolescence stage might have a great impact on the onset and severity of psychiatric diseases in adulthood (Paus et al., 2008; O’Connor and Cryan, 2014). Over the past decade, there has been increasing interest in the potential benefits of early intervention for psychiatric disorders such as depression (Paus et al., 2008; O’Connor and Cryan, 2014; Sarris et al., 2015; Correll et al., 2018).

Preclinical findings suggest that dietary intake of glucoraphanin during juvenile and adolescence can protect against depression-like behaviors after CSDS or LPS administration (Yao et al., 2016b; Zhang et al., 2017b), indicating prophylactic effects of glucoraphanin for depression. Thus, dietary intake of glucoraphanin (or SFN) during juvenile and adolescence might confer stress resilience at adulthood (**Figure 5**). Therefore, it is possible that dietary intake of glucoraphanin (or SFN) during childhood and adolescence stages could prevent the onset of depression in humans during adulthood. Since patients with mood disorder have high relapse rate, dietary intake of glucoraphanin (or SFN) may prevent or minimize relapse from remission, induced by inflammation and/or stress in depressed patients.

## Author Contributions

The author confirms being the sole contributor of this work and approved it for publication.

## Conflict of Interest Statement

The author declares that the research was conducted in the absence of any commercial or financial relationships that could be construed as a potential conflict of interest.

## References

[B1] Abdoulaye I. A. Yi J. G. (2016). A review of recent advances in neuroprotective potential of 3-N-butylphthalide and its derivatives. *Biomed. Res. Int.* 2016:5012341. 10.1155/2016/5012341 28053983 PMC5178327

[B2] Abuelezz S. A. Hendawy N. (2018). Insights into the potential antidepressant mechanisms of cilostazol in chronically restraint rats: impact on the Nrf2 pathway. *Behav. Pharmacol.* 29 28–40. 10.1097/FBP.0000000000000335 28763303

[B3] Al-Jaderi Z. Maghazachi A. A. (2016). Utilization of dimethyl fumarate and related molecules for treatment of multiple sclerosis, cancer, and other diseases. *Front. Immunol.* 7:278. 10.3389/fimmu.2016.00278 27499754 PMC4956641

[B4] Bazinet R. P. Layé S. (2014). Polyunsaturated fatty acids and their metabolites in brain function and disease. *Nat. Rev. Neurosci.* 15 771–785. 10.1038/nrn3820 25387473

[B5] Bouvier E. Brouillard F. Molet J. Claverie D. Cabungcal J. H. Cresto N. (2017). Nrf2-dependent persistent oxidative stress results in stress-induced vulnerability to depression. *Mol. Psychiatry* 22 1701–1713. 10.1038/mp.2016.144 27646262

[B6] Correll C. U. Galling B. Pawar A. Krivko A. Bonetto C. Ruggeri M. (2018). Comparison of early intervention services vs treatment as usual for early-phase psychosis: a systematic review, meta-analysis, and meta-regression. *JAMA Psychiatry* 75 555–565. 10.1001/jamapsychiatry.2018.0623 29800949 PMC6137532

[B7] Dantzer R. O’Connor J. C. Freund G. G. Johnson R. W. Kelley K. W. (2008). From inflammation to sickness and depression: when the immune system subjugates the brain. *Nat. Rev. Neurosci.* 9 46–57. 10.1038/nrn2297 18073775 PMC2919277

[B8] de Zeeuw D. Akizawa T. Audhya P. Bakris G. L. Chin M. Christ-Schmidt H. (2013). Bardoxolone methyl in type 2 diabetes and stage 4 chronic kidney disease. *N. Eng. J. Med.* 369 2492–2503. 10.1056/NEJMoa1306033 24206459 PMC4496027

[B9] Dean B. Tawadros N. Scarr E. Gibbons A. S. (2010). Regionally-specific changes in levels of tumour necrosis factor in the dorsolateral prefrontal cortex obtained postmortem from subjects with major depressive disorder. *J. Affect. Disord.* 120 245–248. 10.1016/j.jad.2009.04.027 19446343

[B10] Dinkova-Kostova A. T. Talalay P. Sharkey J. Zhang Y. Holtzclaw W. D. Wang X. J. (2010). An exceptionally potent inducer of cytoprotective enzymes: elucidation of the structural features that determine inducer potency and reactivity with Keap1. *J. Biol. Chem.* 285 33747–33755. 10.1074/jbc.M110.163485 20801881 PMC2962473

[B11] Dong C. Zhang J. C. Yao W. Ren Q. Yang C. Ma M. (2016). Effects of escitalopram, R-citalopram, and reboxetine on serum levels of tumor necrosis factor-α, interleukin-10, and depression-like behavior in mice after lipopolysaccharide administration. *Pharmacol. Biochem. Behav.* 144 7–12. 10.1016/j.pbb.2016.02.005 26892759

[B12] Dowlati Y. Herrmann N. Swardfager W. Liu H. Sham L. Reim E. K. (2010). A meta-analysis of cytokines in major depression. *Biol. Psychiatry* 67 446–457. 10.1016/j.biopsych.2009.09.033 20015486

[B13] Duman R. S. Monteggia L. M. (2006). A neurotrophic model for stress-related mood disorders. *Biol. Psychiatry* 59 1116–1127. 10.1016/j.biopsych.2006.02.013 16631126

[B14] El-Behadli A. F. Sharp C. Hughes S. O. Obasi E. M. Nicklas T. A. (2015). Maternal depression, stress and feeding styles: towards a framework for theory and research in child obesity. *Br. J. Nutr.* 113 S55–S71. 10.1017/S000711451400333X 25588385

[B15] Fahey J. W. Holtzclaw W. D. Wehage S. L. Wade K. L. Stephenson K. K. Talalay P. (2015). Sulforaphane bioavailability from glucoraphanin-rich broccoli: control by active endogenous myrosinase. *PLoS One* 10:e0140963. 10.1371/journal.pone.0140963 26524341 PMC4629881

[B16] Fahey J. W. Zhang Y. Talalay P. (1997). Broccoli sprouts: an exceptionally rich source of inducers of enzymes that protect against chemical carcinogens. *Proc. Natl. Acad. Sci. U.S.A.* 94 10367–10372. 10.1073/pnas.94.19.10367 9294217 PMC23369

[B17] Golden S. A. Covington H. E. Berton O. Russo S. J. (2011). A standardized protocol for repeated social defeat stress in mice. *Nat. Protoc.* 6 1183–1191. 10.1038/nprot.2011.361 21799487 PMC3220278

[B18] Haapakoski R. Mathieu J. Ebmeier K. P. Alenius H. Kivimäki M. (2015). Cumulative meta-analysis of interleukins 6 and 1β, tumour necrosis factor α and C-reactive protein in patients with major depressive disorder. *Brain Behav. Immun.* 49 206–215. 10.1016/j.bbi.2015.06.001 26065825 PMC4566946

[B19] Hashimoto K. (2009). Emerging role of glutamate in the pathophysiology of major depressive disorder. *Brain Res. Rev.* 61 1056–1123. 10.1016/j.brainresrev.2009.05.005 19481572

[B20] Hashimoto K. (2010). Brain-derived neurotrophic factor as a biomarker for mood disorders: an historical overview and future directions. *Psychiatry Clin. Neurosci.* 64 341–357. 10.1111/j.1440-1819.2010.02113.x 20653908

[B21] Hashimoto K. (2014). Targeting of NMDA receptors in new treatments for schizophrenia. *Expert Opin. Ther. Targets* 18 1049–1063. 10.1517/14728222.2014.934225 24965576

[B22] Hashimoto K. (2015). Inflammatory biomarkers as differential predictors of antidepressant response. *Int. J. Mol. Sci.* 16 7796–7801. 10.3390/ijms16047796 25856677 PMC4425050

[B23] Hashimoto K. Sawa A. Iyo M. (2007). Increased levels of glutamate in brains from patients with mood disorders. *Biol. Psychiatry* 62 1310–1316. 10.1016/j.biopsych.2007.03.017 17574216

[B24] Hashimoto K. Shimizu E. Iyo M. (2004). Critical role of brain-derived neurotrophic factor in mood disorders. *Brain Res. Rev.* 45 104–114. 10.1016/j.brainresrev.2004.02.003 15145621

[B25] Honda T. Sundararajan C. Yoshizawa H. Su X. Honda Y. Liby K. T. (2007). Novel tricyclic compounds having acetylene groups at C-8a and cyano enones in rings A and C: highly potent anti-inflammatory and cytoprotective agents. *J. Med. Chem.* 50 1731–1734. 10.1021/jm070141c 17367124 PMC2522370

[B26] Honda T. Yoshizawa H. Sundararajan C. David E. Lajoie M. J. Favaloro F. G. Jr. (2011). Tricyclic compounds containing nonenolizable cyano enones: a novel class of highly potent anti-inflammatory and cytoprotective agents. *J. Med. Chem.* 54 1762–1778. 10.1021/jm101445p 21361338 PMC3251033

[B27] Hsu M. C. Tung C. Y. Chen H. E. (2018). Omega-3 polyunsaturated fatty acid supplementation in prevention and treatment of maternal depression: putative mechanism and recommendation. *J. Affect. Disord.* 238 47–61. 10.1016/j.jad.2018.05.018 29860183

[B28] Jia J. Wei C. Liang J. Zhou A. Zuo X. Song H. (2016). The effects of DL-3-n-butylphthalide in patients with vascular cognitive impairment without dementia caused by subcortical ischemic small vessel disease: a multicentre, randomized, double-blind, placebo-controlled trial. *Alzheimers Dement.* 12 89–99. 10.1016/j.jalz.2015.04.010 26086183

[B29] Kensler T. W. Egner P. A. Agyeman A. S. Visvanathan K. Groopman J. D. Chen J. G. (2013). Keap1-Nrf2 signaling: a target for cancer prevention by sulforaphane. *Top Curr. Chem.* 329 163–177. 10.1007/128_2012_339 22752583 PMC3553557

[B30] Kobayashi E. Suzuki T. Yamamoto M. (2013). Roles Nrf2 plays in myeloid cells and related disorders. *Oxid. Med. Cell. Longev.* 2013:529219. 10.1155/2013/529219 23819012 PMC3684031

[B31] Köhler C. A. Freitas T. H. Stubbs B. Maes M. Solmi M. Veronese N. (2018). Peripheral alterations in cytokine and chemokine levels after antidepressant drug treatment for major depressive disorder: systematic review and meta-analysis. *Mol. Neurobiol.* 55 4195–4206. 10.1007/s12035-017-0632-1 28612257

[B32] Kostov R. V. Knatko E. V. McLaughlin L. A. Henderson C. J. Zheng S. Huang J. T. (2015). Pharmacokinetics and pharmacodynamics of orally administered acetylenic tricyclic bis(cyanoenone), a highly potent Nrf2 activator with a reversible covalent mode of action. *Biochem. Biophys. Res. Commun.* 465 402–407. 10.1016/j.bbrc.2015.08.016 26265043 PMC4567061

[B33] Krishnan V. Nestler E. J. (2008). The molecular neurobiology of depression. *Nature* 455 894–902. 10.1038/nature07455 18923511 PMC2721780

[B34] Kwak M. K. Kensler T. W. (2010). Targeting NRF2 signaling for cancer chemoprevention. *Toxicol. Appl. Pharmacol.* 244 66–76. 10.1016/j.taap.2009.08.028 19732782 PMC3584341

[B35] Lai J. S. Hiles S. Bisquera A. Hure A. J. McEvoy M. Attia J. (2014). A systematic review and meta-analysis of dietary patterns and depression in community-dwelling adults. *Am. J. Clin. Nutr.* 99 181–197. 10.3945/ajcn.113.069880 24196402

[B36] Li S. Yang C. Fang X. Zhan G. Huang N. Gao J. (2018). Role of Keap1-Nrf2 signaling in anhedonia symptoms in a rat model of chronic neuropathic pain: improvement with sulforaphane. *Front. Pharmacol.* 9:887. 10.3389/fphar.2018.00887 30135655 PMC6092692

[B37] Lin P. Y. Chang C. H. Chong M. F. Chen H. Su K. P. (2017). Polyunsaturated fatty acids in perinatal depression: a systematic review and meta-analysis. *Biol. Psychiatry* 82 560–569. 10.1016/j.biopsych.2017.02.1182 28410627

[B38] Lin P. Y. Huang S. Y. Su K. P. (2010). A meta-analytic review of polyunsaturated fatty acid compositions in patients with depression. *Biol. Psychiatry* 68 140–147. 10.1016/j.biopsych.2010.03.018 20452573

[B39] Liu X. Yan Y. Li F. Zhang D. (2016). Fruit and vegetable consumption and the risk of depression: a meta-analysis. *Nutrition* 32 296–302. 10.1016/j.nut.2015.09.009 26691768

[B40] Lu X. H. Dwyer D. S. (2005). Second-generation antipsychotic drugs, olanzapine, quetiapine, and clozapine enhance neurite outgrowth in PC12 cells via PI3K/AKT, ERK, and pertussis toxin-sensitive pathways. *J. Mol. Neurosci.* 27 43–64. 10.1385/JMN:27:1:043 16055946

[B41] Lukic I. Mitic M. Djordjevic J. Tatalovic N. Bozovic N. Soldatovic I. (2014). Lymphocyte levels of redox-sensitive transcription factors and antioxidative enzymes as indicators of pro-oxidative state in depressive patients. *Neuropsychology* 70 1–9. 10.1159/000362841 25170744

[B42] Lynch R. Diggins E. L. Connors S. L. Zimmerman A. W. Singh K. Liu H. (2017). Sulforaphane from broccoli reduces symptoms of autism: a follow-up case series from a randomized double-blind study. *Glob. Adv. Health Med.* 6:21644957X17735826. 10.1177/2164957X17735826 29147630 PMC5672987

[B43] Ma Q. (2013). Role of Nrf2 in oxidative stress and toxicity. *Annu. Rev. Pharmacol. Toxicol.* 53 401–426. 10.1146/annurev-pharmtox-011112-140320 23294312 PMC4680839

[B44] Martín-de-Saavedra M. D. Budni J. Cunha M. P. Gómez-Rangel V. Lorrio S. Del Barrio L. (2013). Nrf2 participates in depressive disorders through an anti-inflammatory mechanism. *Psychoneuroendocrinology* 38 2010–2022. 10.1016/j.psyneuen.2013.03.020 23623252

[B45] Martín-Hernández D. Caso J. R. Javier Meana J. Callado L. F. Madrigal J. L. M. García-Bueno B. (2018). Intracellular inflammatory and antioxidant pathways in postmortem frontal cortex of subjects with major depression: effect of antidepressants. *J. Neuroinflammation* 15:251. 10.1186/s12974-018-1294-2 30180869 PMC6122627

[B46] Marzec J. M. Christie J. D. Reddy S. P. Jedlicka A. E. Vuong H. Lanken P. N. (2007). Functional polymorphisms in the transcription factor NRF2 in humans increase the risk of acute lung injury. *FASEB J.* 21 2237–2246. 10.1096/fj.06-7759com 17384144

[B47] Matsuura A. Ishima T. Fujita Y. Iwayama Y. Hasegawa S. Kawahara-Miki R. (2018). Dietary glucoraphanin prevents the onset of psychosis in the adult offspring after maternal immune activation. *Sci. Rep.* 8:2158. 10.1038/s41598-018-20538-3 29391571 PMC5794794

[B48] McCullumsmith R. E. Hammond J. H. Shan D. Meador-Woodruff J. H. (2013). Postmortem brain: an underutilized substrate for studying severe mental illness. *Neuropsychopharmacology* 39 65–87. 10.1038/npp.2013.239 24091486 PMC3857666

[B49] Mechawar N. Savitz J. (2016). Neuropathology of mood disorders: do we see the stigmata of inflammation? *Transl. Psychiatry* 6:e946. 10.1038/tp.2016.212 27824355 PMC5314124

[B50] Mello A. H. Gassenferth A. Souza L. R. Fortunato J. J. Rezin G. T. (2014). ω-3 and major depression: a review. *Acta Neuropsychiatr.* 26 178–185. 10.1017/neu.2013.52 25142194

[B51] Mellon S. H. Wolkowitz O. M. Schonemann M. D. Epel E. S. Rosser R. Burke H. B. (2016). Alterations in leukocyte transcriptional control pathway activity associated with major depressive disorder and antidepressant treatment. *Transl. Psychiatry* 6:e821. 10.1038/tp.2016.79 27219347 PMC5070063

[B52] Mendez-David I. Tritschler L. Ali Z. E. Damiens M. H. Pallardy M. David D. J. (2015). Nrf2-signaling and BDNF: a new target for the antidepressant-like activity of chronic fluoxetine treatment in a mouse model of anxiety/depression. *Neurosci. Lett.* 597 121–126. 10.1016/j.neulet.2015.04.036 25916883

[B53] Miller A. H. Haroon E. Felger J. C. (2017). Therapeutic implications of brain-immune interactions: treatment in translation. *Neuropsychopharmacology* 42 334–359. 10.1038/npp.2016.167 27555382 PMC5143492

[B54] Miller A. H. Maletic V. Raison C. L. (2009). Inflammation and its discontents: the role of cytokines in the pathophysiology of major depression. *Biol. Psychiatry* 65 732–741. 10.1016/j.biopsych.2008.11.029 19150053 PMC2680424

[B55] Miller A. H. Raison C. L. (2016). The role of inflammation in depression: from evolutionary imperative to modern treatment target. *Nat. Rev. Immunol.* 16 22–34. 10.1038/nri.2015.5 26711676 PMC5542678

[B56] Mills E. A. Ogrodnik M. A. Plave A. Mao-Draayer Y. (2018). Emerging understanding of the mechanism of action for dimethyl fumarate in the treatment of multiple sclerosis. *Front. Neurol.* 9:5. 10.3389/fneur.2018.00005 29410647 PMC5787128

[B57] Murakami K. Sasaki S. (2010). Dietary intake and depressive symptoms: a systemic review of observational studies. *Mol. Nutr. Food. Res.* 54 471–488. 10.1002/mnfr.200900157 19998381

[B58] Nestler E. J. Barrot M. DiLeone R. J. Eisch A. J. Gold S. J. Monteggia L. M. (2002). Neurobiology of depression. *Neuron* 34 13–25. 10.1016/S0896-6273(02)00653-0 11931738

[B59] Nestler E. J. Hyman S. E. (2010). Animal models of neuropsychiatric disorders. *Nat. Rev. Neurosci.* 13 1161–1169. 10.1038/nn.2647 20877280 PMC3750731

[B60] O’Connell M. A. Hayes J. D. (2015). The Keap1/Nrf2 pathway in health and disease: from the bench to the clinic. *Biochem. Soc. Trans.* 43 687–689. 10.1042/BST20150069 26551713

[B61] O’Connor J. C. Lawson M. A. André C. Moreau M. Lestage J. Castanon N. (2009). Lipopolysaccharide-induced depressive-like behavior is mediated by indoleamine 2,3-dioxygenase activation in mice. *Mol. Psychiatry* 14 511–522. 10.1038/sj.mp.4002148 18195714 PMC2683474

[B62] O’Connor R. M. Cryan J. F. (2014). Adolescent brain vulnerability and psychopathology through the generations: role of diet and dopamine. *Biol. Psychiatry* 75 4–6. 10.1016/j.biopsych.2013.10.022 24314061

[B63] Ohgi Y. Futamura T. Kikuchi T. Hashimoto K. (2013). Effects of antidepressants on alternations in serum cytokines and depressive-like behavior in mice after lipopolysaccharide administration. *Pharmacol. Biochem. Behav.* 103 853–859. 10.1016/j.pbb.2012.12.003 23262300

[B64] Opie A. O’Neil R. S. Itsiopoulos C. Jacka F. N. (2015). The impact of whole-of-diet interventions on depression and anxiety: a systematic review of randomised controlled trials. *Public Health Nutr.* 18 2074–2093. 10.1017/S1368980014002614 25465596 PMC10271872

[B65] Paus T. Keshavan M. Giedd J. N. (2008). Why do many psychiatric disorders emerge during adolescence? *Nat. Rev. Neurosci.* 9 947–957. 10.1038/nrn2513 19002191 PMC2762785

[B66] Pergola P. E. Raskin P. Toto R. D. Meyer C. J. Huff J. W. Grossman E. B. (2011). Bardoxolone methyl and kidney function in CKD with type 2 diabetes. *N. Eng. J. Med.* 365 327–336. 10.1056/NEJMoa1105351 21699484

[B67] Raison C. L. Lowry C. A. Rook G. A. (2010). Inflammation, sanitation, and consternation: loss of contact with coevolved, tolerogenic microorganisms and the pathophysiology and treatment of major depression. *Arch. Gen. Psychiatry* 67 1211–1224. 10.1001/archgenpsychiatry.2010.161 21135322 PMC3724429

[B68] Ren D. Villeneuve N. F. Jiang T. Wu T. Lau A. Toppin H. A. (2011). Brusatol enhances the efficacy of chemotherapy by inhibiting the Nrf2-mediated defense mechanism. *Proc. Natl. Acad. Sci. U.S.A.* 108 1433–1438. 10.1073/pnas.1014275108 21205897 PMC3029730

[B69] Saghafian F. Malmir H. Saneei P. Milajerdi A. Larijani B. Esmaillzadeh A. (2018). Fruit and vegetable consumption and risk of depression: accumulative evidence from an updated systematic review and meta-analysis of epidemiological studies. *Br. J. Nutr.* 119 1087–1101. 10.1017/S0007114518000697 29759102

[B70] Sarris J. Logan A. C. Akbaraly T. N. Amminger G. P. Balanzá-Martínez V. Freeman M. P. (2015). Nutritional medicine as mainstream in psychiatry. *Lancet Psychiatry* 2 271–274. 10.1016/S2215-0366(14)00051-0 26359904

[B71] Sedlak T. W. Nucifora L. G. Koga M. Shaffer L. S. Higgs C. Tanaka T. (2018). Sulforaphane augments glutathione and influences brain metabolites in human subjects: a clinical pilot study. *Mol. Neuropsychiatry* 3 314–222. 10.1159/000487639 29888232 PMC5981770

[B72] Shelton R. C. Claiborne J. Sidoryk-Wegrzynowicz M. Reddy R. Aschner M. Lewis D. A. (2011). Altered expression of genes involved in inflammation and apoptosis in frontal cortex in major depression. *Mol. Psychiatry* 16 751–762. 10.1038/mp.2010.52 20479761 PMC2928407

[B73] Shiina A. Kanahara N. Sasaki H. Oda Y. Hashimoto T. Hasegawa T. (2015). An open study of sulforaphane-rich broccoli sprout extract in patients with schizophrenia. *Clin. Psychopharmacol. Neurosci.* 13 62–67. 10.9758/cpn.2015.13.1.62 25912539 PMC4423155

[B74] Shirai Y. Fujita Y. Hashimoto R. Ohi K. Yamamori H. Yasuda Y. (2015). Dietary intake of sulforaphane-rich broccoli sprout extracts during juvenile and adolescence can prevent phencyclidine-induced cognitive deficits at adulthood. *PLoS One* 10:e0127244. 10.1371/journal.pone.0127244 26107664 PMC4479552

[B75] Singh A. Venkannagari S. Oh K. H. Zhang Y. Q. Rohde J. M. Liu L. (2016). Small molecule inhibitor of NRF2 selectively intervenes therapeutic resistance in KEAP1-deficient NSCLC tumors. *ACS Chem. Biol.* 11 3214–3225. 10.1021/acschembio.6b00651 27552339 PMC5367156

[B76] Singh K. Connors S. L. Macklin E. A. Smith K. D. Fahey J. W. Talalay P. (2014). Sulforaphane treatment of autism spectrum disorder (ASD). *Proc. Natl. Acad. Sci. U.S.A.* 111 15550–15555. 10.1073/pnas.1416940111 25313065 PMC4217462

[B77] Suzuki T. Motohashi H. Yamamoto M. (2013a). Toward clinical application of the Keap1-Nrf2 pathway. *Trends Pharmacol. Sci.* 34 340–346. 10.1016/j.tips.2013.04.005 23664668

[B78] Suzuki T. Shibata T. Takaya K. Shiraishi K. Kohno T. Kunitoh H. (2013b). Regulatory nexus of synthesis and degradation deciphers cellular Nrf2 expression levels. *Mol. Cell. Biol.* 33 2402–2412. 10.1128/MCB.00065-13 23572560 PMC3700104

[B79] Suzuki T. Yamamoto M. (2015). Molecular basis of the Keap1-Nrf2 system. *Free Radic. Biol. Med.* 88 93–100. 10.1016/j.freeradbiomed.2015.06.006 26117331

[B80] Tsuchida K. Tsujita T. Hayashi M. Ojima A. Keleku-Lukwete N. Katsuoka F. (2017). Halofuginone enhances the chemo-sensitivity of cancer cells by suppressing NRF2 accumulation. *Free Radic. Biol. Med.* 103 236–247. 10.1016/j.freeradbiomed.2016.12.041 28039084

[B81] Wang X. Zhao F. Wang X. Niu Y. Niu L. Wang C. (2018). Recent advances in nutrition for the treatment of depressive disorder. *Curr. Pharm. Des.* 10.2174/1381612824666180803113106 [Epub ahead of print]. 30073920

[B82] Wang Y. Y. Yang Y. X. Zhe H. He Z. X. Zhou S. F. (2014). Bardoxolone methyl (CDDO-Me) as a therapeutic agent: an update on its pharmacokinetic and pharmacodynamic properties. *Drug Des. Devel. Ther.* 8 2075–2088. 10.2147/DDDT.S68872 25364233 PMC4211867

[B83] Wardyn J. D. Ponsford A. H. Sanderson C. M. (2015). Dissecting molecular cross-talk between Nrf2 and NF-κB response pathways. *Biochem. Soc. Trans.* 43 621–626. 10.1042/BST20150014 26551702 PMC4613495

[B84] Williams B. Dwyer D. S. (2009). Structure-based discovery of low molecular weight compounds that stimulate neurite outgrowth and substitute for nerve growth factor. *J. Neurochem.* 110 1876–1884. 10.1111/j.1471-4159.2009.06291.x 19627449 PMC2753211

[B85] World Health Organization [WHO] (2017). *Depression.* Available at: http://www.who.int/en/news-room/fact-sheets/detail/depression

[B86] Yamamoto M. Kensler T. W. Motohashi H. (2018). The KEAP1-NRF2 system: a thiol-based sensor-effector apparatus for maintaining redox homeostasis. *Physiol. Rev.* 98 1169–1203. 10.1152/physrev.00023.2017 29717933 PMC9762786

[B87] Yamamoto T. Kyo M. Kamiya T. Tanaka T. Engel J. D. Motohashi H. (2006). Predictive base substitution rules that determine the binding and transcriptional specificity of Maf recognition elements. *Genes Cells* 11 575–591. 10.1111/j.1365-2443.2006.00965.x 16716189

[B88] Yang B. Ren Q. Zhang J. C. Chen Q. X. Hashimoto K. (2017). Altered expression of BDNF, BDNF pro-peptide and their precursor proBDNF in brain and liver tissues from psychiatric disorders: rethinking the brain-liver axis. *Transl. Psychiatry* 7:e1128. 10.1038/tp.2017.95 28509900 PMC5534963

[B89] Yang B. Yang C. Ren Q. Zhang J. C. Chen Q. X. Shirayama Y. (2016). Regional differences in the expression of brain-derived neurotrophic factor (BDNF) pro-peptide, proBDNF and preproBDNF in the brain confer stress resilience. *Eur. Arch. Psychiatry Clin. Neurosci.* 266 765–769. 10.1007/s00406-016-0693-6 27094192

[B90] Yang C. Cheng Y. Zhao J. Rong J. (2015c). Releasing Nrf2 to promote neurite outgrowth. *Neural. Regen. Res.* 10 1934–1935. 10.4103/1673-5374.169618 26889175 PMC4730811

[B91] Yang C. Shirayama Y. Zhang J. C. Ren Q. Hashimoto K. (2015a). Peripheral interleukin-6 promotes resilience versus susceptibility to inescapable stress. *Acta Neuropsychiatr.* 27 312–316. 10.1017/neu.2015.36 26017899

[B92] Yang C. Shirayama Y. Zhang J. C. Ren Q. Hashimoto K. (2015b). Regional differences in brain-derived neurotrophic factor levels and dendritic spine density confer resilience to inescapable stress. *Int. J. Neuropsychopharmacol.* 18:yu121. 10.1093/ijnp/pyu121 25568287 PMC4540100

[B93] Yang M. Dang R. Xu P. Guo Y. Han W. Liao D. (2018). Dl-3-n-Butylphthalide improves lipopolysaccharide-induced depressive-like behavior in rats: involvement of Nrf2 and NF-κB pathways. *Psychopharmacology* 235 2573–2585. 10.1007/s00213-018-4949-x 29943092

[B94] Yao W. Zhang J. C. Dong C. Zhuang C. Hirota S. Inanaga K. (2015). Effects of amycenone on serum levels of tumor necrosis factor-α, interleukin-10, and depression-like behavior in mice after lipopolysaccharide administration. *Pharmacol. Biochem. Behav.* 136 7–12. 10.1016/j.pbb.2015.06.012 26150007

[B95] Yao W. Zhang J. W. Ishima T. Dong C. Yang C. Ren Q. (2016a). Antidepressant effects of TBE-31 and MCE-1, the novel Nrf2 activators, in an inflammation model of depression. *Eur. J. Pharmacol.* 793 21–27. 10.1016/j.ejphar.2016.10.037 27815170

[B96] Yao W. Zhang J. W. Ishima T. Dong C. Yang C. Ren Q. (2016b). Role of Keap1-Nrf2 signaling in depression and dietary intake of glucoraphanin confers stress resilience in mice. *Sci. Rep.* 6:30659. 10.1038/srep30659 27470577 PMC4965765

[B97] Young J. J. Bruno D. Pomara N. (2014). A review of the relationship between pro-inflammatory cytokines and major depressive disorder. *J. Affect. Disord.* 169 15–20. 10.1016/j.jad.2014.07.032 25128861

[B98] Yu C. Pan S. Dong M. Niu Y. (2017). Astragaloside IV attenuates lead acetate-induced inhibition of neurite outgrowth through activation of Akt-dependent Nrf2 pathway in vitro. *Biochem. Biophys. Acta* 1863 1195–1203. 10.1016/j.bbadis.2017.03.006 28315454

[B99] Zhang J. C. Wu J. Fujita Y. Yao W. Ren Q. Yang C. (2014). Antidepressant effects of TrkB ligands on depression-like behavior and dendritic changes in mice after inflammation. *Int. J. Neuropsychopharmacol.* 18:yu077. 10.1093/ijnp/pyu077 25628381 PMC4360225

[B100] Zhang J. C. Yao W. Dong C. Han M. Shirayama Y. Hashimoto K. (2018). Keap1-Nrf2 signaling pathway confers resilience versus susceptibility to inescapable electric stress. *Eur. Arch. Psychiatry Clin. Neurosci.* 10.1007/s00406-017-0848-0 [Epub ahead of print]. 29119264

[B101] Zhang J. C. Yao W. Dong C. Yang C. Ren Q. Ma M. (2017a). Blockade of interleukin-6 receptor in the periphery promotes rapid and sustained antidepressant actions: a possible role of gut-microbiota-brain axis. *Transl. Psychiatry* 7:e1138. 10.1038/tp.2017.112 28556833 PMC5534942

[B102] Zhang J. C. Yao W. Dong C. Yang C. Ren Q. Ma M. (2015). Comparison of ketamine, 7,8-dihydroxyflavone, and ANA-12 antidepressant effects in the social defeat stress model of depression. *Psychopharmacology* 232 4325–4335. 10.1007/s00213-015-4062-3 26337614

[B103] Zhang J. C. Yao W. Dong C. Yang C. Ren Q. Ma M. (2017b). Prophylactic effects of sulforaphane on depression-like behavior and dendritic changes in mice after inflammation. *J. Nutr. Biochem.* 39 134–144. 10.1016/j.jnutbio.2016.10.004 27833054

[B104] Zhang J. C. Yao W. Hashimoto K. (2016). Brain-derived neurotrophic factor (BDNF) – TrkB signaling in inflammation-related depression and potential therapeutic targets. *Curr. Neuropharmacol.* 14 721–731. 10.2174/1570159X14666160119094646 26786147 PMC5050398

[B105] Zhang Y. Talalay P. Cho C. G. Posner G. H. (1992). A major inducer of anticarcinogenic protective enzymes from broccoli: isolation and elucidation of structure. *Proc. Natl. Acad. Sci. U.S.A.* 89 2399–2403. 10.1073/pnas.89.6.2399 1549603 PMC48665

